# Recent Advances in THz Detection of Water

**DOI:** 10.3390/ijms241310936

**Published:** 2023-06-30

**Authors:** Hongyi Ge, Zhenyu Sun, Yuying Jiang, Xuyang Wu, Zhiyuan Jia, Guangyuan Cui, Yuan Zhang

**Affiliations:** 1 Key Laboratory of Grain Information Processing & Control, Ministry of Education, Henan University of Technology, Zhengzhou 450001, China; gehongyi2004@163.com (H.G.); sunzhenyu_0523@163.com (Z.S.); wuxuyang1217@163.com (X.W.); jiazhiyuan@stu.haut.edu.cn (Z.J.); m15239599850_2@163.com (G.C.); zy_haut@163.com (Y.Z.); 2 Henan Provincial Key Laboratory of Grain Photoelectric Detection and Control, Zhengzhou 450001, China; 3 College of Information Science and Engineering, Henan University of Technology, Zhengzhou 450001, China; 4 School of Artificial Intelligence and Big Data, Henan University of Technology, Zhengzhou 450001, China

**Keywords:** terahertz waves, moisture content, distribution, theoretical model, water molecule network

## Abstract

The frequency range of terahertz waves (THz waves) is between 0.1 and 10 THz and they have properties such as low energy, penetration, transients, and spectral fingerprints, which are especially sensitive to water. Terahertz, as a frontier technology, have great potential in interpreting the structure of water molecules and detecting biological water conditions, and the use of terahertz technology for water detection is currently frontier research, which is of great significance. Firstly, this paper introduces the theory of terahertz technology and summarizes the current terahertz systems used for water detection. Secondly, an overview of theoretical approaches, such as the relaxation model and effective medium theory related to water detection, the relationship between water molecular networks and terahertz spectra, and the research progress of the terahertz detection of water content and water distribution visualization, are elaborated. Finally, the challenge and outlook of applications related to the terahertz wave detection of water are discussed. The purpose of this paper is to explore the research domains on water and its related applications using terahertz technology, as well as provide a reference for innovative applications of terahertz technology in moisture detection.

## 1. Introduction

Water, named as the fountain of life, exhibits different structures and functions in molecular cytology. As an important part of cells, it is involved in almost all life activities and exists in all plants and animals. Two-thirds of human tissues are water, and a sufficient water intake is needed every day to ensure the smooth circulation of blood and lymphatic fluid and maintain life. Moreover, water is also an important component of plant physiological processes and biological tissues, which are involved in photosynthesis, hydrolysis, transpiration and the nutrient transport of plants, and thus water is an important indicator to evaluate the growth status of plants [1]. The moisture content in grain cereals affects their quality to some extent: specifically, too much moisture can cause mold, insect damage, and temperature changes, while too little moisture can destroy their organic matter and then affect grain quality [2]. Therefore, the detection of moisture conditions is extremely important.

Traditional moisture detection methods are divided into destructive and nondestructive detection. Destructive detection methods include the drying method, chemical method, etc.; nondestructive detection methods include the resistance method, capacitance method, etc. [3]. However, these methods are destructive and noncontinuous, waste time, etc., which makes it difficult to meet the demand of rapid nondestructive detection. With the development of spectroscopy technology, hyperspectral, Fourier transform infrared and Raman spectroscopy [4] are widely used for moisture content detection, but these methods usually need to isolate the range of water-sensitive features within the radiation spectrum and are subject to interference from environmental factors. The detection accuracy of Raman spectroscopy [5] is high, but its scattering signal is weak, which makes its detection depth limited, and it can only detect the surface water, while it is easily interfered with by the background signal. The resolution of Fourier transform infrared spectroscopy [6] is high, but its absorption characteristics of water are weak, and it has certain limitations for the detection of low concentrations of moisture. Therefore, a more efficient method for moisture detection is needed.

Terahertz waves are electromagnetic waves with frequencies in the range of 0.1–10 THz (wavelength of 3000–30 μm), between microwave and infrared light, and are the transition zone from electronics to photonics in a macroscopic sense [7]. Terahertz waves are characterized by high penetration, low energy, water absorption, and transients [8,9], and terahertz technology is a very important cross-cutting frontier field that is widely used in radar, remote sensing, medical diagnosis, food safety detection, etc. It can effectively promote national technological innovation and economic development, and it has significant applications for the national economy and national security. Water is extremely sensitive to terahertz waves and has a strong absorption effect on terahertz waves [10], and the reason is that water molecules are polar molecules, which are connected to nearby water molecules through four hydrogen bonds [11]. When interacting with terahertz waves, the hydrogen bonds in the water network resonate by their action, and the water molecules form a new hydrogen bond network by relaxation; the relaxation time occurs in picosecond and sub-picosecond magnitudes [12]. While interacting with terahertz, an extremely strong absorption effect occurs. In addition, terahertz technology combined with the relaxation model, effective medium theory model and machine learning and other related theories can achieve an effective detection of moisture. The terahertz detection of water is widely used in areas such as biomedical imaging, environmental monitoring, and industrial processes. Stanley Sy et al. [13] used terahertz reflectance spectroscopy measurements on healthy and cirrhotic tissue to investigate the correlation among terahertz properties, moisture content, structural changes, and cirrhosis. Borovkova et al. [14] combined THz-TDS with the LLL model to measure the moisture content in pork muscle. The results showed that the mean error was much lower than the changes in moisture content observed in lesions and tumors relative to normal tissue. Song et al. [15] detected the high-temperature water vapor in industrial processes using the continuous-wave terahertz technique. It showed that the linear relationship between the absorption peak area and the water vapor volume mixing ratio (VMR) at three absorption peaks (0.557 THz, 0.658 THz, and 0.752 THz) match HITRAN-based simulations closely. Fan et al. [16] used terahertz spectroscopy for monitoring water vapor in a natural gas pipeline in combination with the proposed pressure-gradient-based method to capture the transmitted terahertz signal at two different absolute pressures. The results showed that water vapor of at least 62 ppm can be detected at 100 bar with a path length of 14.7 cm. This review introduces the THz spectral imaging technique for moisture detection, the Debye relaxation model, and other related theories, reviews the research progress of the terahertz wave detection of water, and finally discusses the challenges faced by the terahertz wave detection of water and gives a positive outlook, which provides innovative values for the application of terahertz technology in the field of moisture detection.

## 2. Related Technical Theories

### 2.1. Terahertz Spectroscopy and Imaging

With the development of information technology, terahertz technology has become the focus of research in recent years. Located in the transition zone between macroscopic electronics and microscopic photonics, terahertz can neither be fully explained by optical theory nor by electronics theory [17]. Terahertz are widely used in various fields with the help of their unique properties, such as high penetration and low energy. Terahertz spectroscopy contains a large amount of physical and chemical information of the tested sample and can reflect the van der Waals forces, dipole vibrational jumps, and other states of water molecules through the interaction between water molecules and terahertz waves [18]. The processing of terahertz spectra in combination with chemometric methods enables the terahertz spectra to correctly characterize the tested sample [19]. Terahertz imaging is implemented on the basis of terahertz spectroscopy by scanning and imaging the tested sample, and each pixel point of the obtained image contains spectral information; compared with other imaging methods, terahertz imaging has more advantages and contains more characteristic information [20].

The acquisition of terahertz spectra includes both the transmitter and the detector side of the terahertz pulses, which are generated by the terahertz transmitter side to irradiate the sample, which in turn transports the transmitted or reflected signal to the detector side to acquire a terahertz time-domain spectrum containing information about the sample [21]. Terahertz radiation can be divided into electronic and photonic terahertz emission sources according to the mode of generation.

The electronically based terahertz source generation methods include semiconductor terahertz quantum cascade lasers and terahertz free-electron lasers. Quantum cascade lasers are unipolar lasers in which only electrons are involved [22]. The electrons jump from a higher energy state to a lower energy state and emit photons, resulting in terahertz radiation. The free-electron laser uses a magnetic oscillator device consisting of electrons moving at a high speed through a periodic magnetic field, and a cavity mirror is installed at both ends so that the electromagnetic waves radiated by the electrons form a resonance in the cavity, and the free electrons undergo excited radiation, resulting in terahertz laser output [23].

Generation methods of photonics terahertz sources mainly include photoconductive antennas and optical rectification [24]. The photoconductive antenna consists of a semiconductor substrate and an electrode in contact with it. The role of the electrodes are to apply a bias electric field to the semiconductor material between the electrodes, while its structure can be designed to improve the radiation efficiency. An ultrashort laser beam is focused onto the semiconductor material between the electrodes, and if the laser photon energy is larger than the energy gap width of the semiconductor substrate material, electrons can be excited to the conduction band, forming electron–hole pairs, i.e., photogenerated free carriers. These photogenerated free carriers form a transient changing current under the action of a bias electric field. From Maxwell’s equations, it is known that the change of current causes electromagnetic field radiation. This electromagnetic radiation is located in the terahertz band when the pulse width of the ultrashort laser is in the sub-picosecond order or below and the carrier lifetime of the semiconductor material is short enough [25]. Optical rectification is a second-order nonlinear process. When the optical field interacts with a medium (nonlinear electro-optical crystal) with second-order nonlinear properties, a terahertz wave is generated by the optical differential frequency of two photons of different frequencies within the spectrum of the femtosecond laser pulse. The laser pulse energy directly affects the THz wave energy, and the conversion efficiency depends mainly on the nonlinear coefficient and phase matching conditions [26].

**Figure 1:**
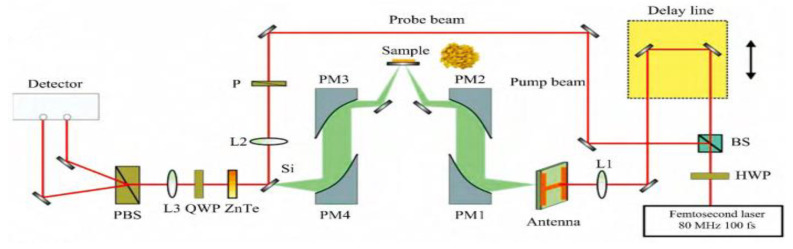
Schematic diagram of THz-TDS system structure [29].

A typical terahertz time-domain spectroscopy system (THz-TDS) is shown in Figure 1. The femtosecond laser is divided into a stronger pump light and a weaker detection light through a beam splitter. The pump light is incident on the transmitting crystal via a delay device, and a terahertz pulse is generated by the time-resolved photoluminescence technique [27]. This terahertz wave is focused by a terahertz lens set and then irradiated onto the detection target, and the reflected or transmitted terahertz wave is received by a terahertz receiver device. Another detection beam is transmitted to the terahertz receiver device through a series of reflectors to generate photogenerated carriers, which generate a current signal under the action of terahertz waves. Two pulses are used to probe the sample information via the pump–probe technique [28]. By adjusting the relative time delay of the terahertz pulse and the detection light through the time delay device, the intensity of the current signal corresponding to the electric field of the terahertz wave at different moments can be obtained, and the time waveform of the terahertz pulse can be reconstructed by detecting the intensity change in the current signal output from the terahertz detector with a time delay [29].

The time domain information of the spectrum is obtained directly by testing the sample using terahertz equipment, and the frequency domain information can be obtained by fast Fourier transform (FFT), in which the variation of the amplitude and phase of the terahertz wave can be obtained [30]. Optical parameters, such as the absorption coefficient [31], dielectric constant, refractive index [32], and transmittance of the sample, can be obtained from the frequency domain information using specific optical formulas [33]. According to the data processing model of Fresnel’s formula [34], the sample refractive index *n(ω)* and the absorption coefficient *α(ω)* can be calculated by the following equations:

(1) $$n\left(\omega \right)=\frac{\emptyset \left(\omega \right)}{\omega d}+1$$

(2) $$\alpha (\omega )=\frac{2k(\omega )\omega }{c}=\frac{2}{d}\ln\frac{4n(\omega )}{{A(\omega )(n\left(\omega \right)+1)}^{2}}$$

where *ω* is the frequency; c is the speed of light in a vacuum; *d* is the thickness of the sample; *A(ω)* denotes the amplitude ratio; and ∅*(ω)* stands for the phase difference between the sample signal and the reference signal, respectively.

Based on the presence or absence of radiation sources, terahertz imaging techniques are divided into active and passive imaging techniques [35], with passive imaging techniques mostly using quasi-optical techniques and active imaging mostly using radar techniques or time-domain spectroscopy.

Based on the terahertz time-domain spectroscopy theory, terahertz scanning imaging includes transmission scanning and reflection scanning, and the terahertz two-dimensional intensity image of the sample is acquired by scanning each pixel point [36]. With the development of terahertz imaging technology, the imaging methods have become diverse, including TDS, chromatography, near-field, and continuous-wave imaging. Terahertz time-domain spectral pulse imaging technology uses ultrafast terahertz time-domain pulses to achieve an ultra-high resolution in the distance direction, usually combined with two-dimensional scanning and quasi-optical focusing to achieve a lateral resolution [37]. The key technologies include the femtosecond laser, photoconductive antenna, delay line, and time-domain signal processing technology, which are mainly used in the field of nondestructive testing.

A partial comparison of terahertz imaging techniques is shown in Table 1, where the different imaging modalities differ in terms of key technologies and application areas. Among them, terahertz systems with higher imaging resolutions and faster imaging speeds are more beneficial for the assessment of the water status of organisms.

Some of the mature terahertz systems currently used for moisture detection are shown in Table 2, and these methods are suitable for different experimental environments depending on the detection scheme, frequency range, and data processing methods.

### 2.2. Debye Relaxation Model

Relaxation is the process by which a system is perturbed into a nonequilibrium state due to changes in the environment, and it returns to equilibrium from a deviation from equilibrium, where the microscopic particles in the system interact to produce an energy exchange [49]. The laws of relaxation are related to the nature of interactions among microscopic particles, and we can better understand the interactions within water molecules by studying the relaxation process of water [50].

Water, as a polar liquid, has a dielectric relaxation phenomenon in the terahertz band where its refractive index and dielectric constant decrease with an increasing frequency [51]. Debye proposed the relaxation theory based on this phenomenon, which treats the molecule as a continuous dielectric sphere with macroscopic viscosity that can be described by a viscous damping model. The Debye equation is shown as follows [52]:

(3) $$\hat{\varepsilon }=\varepsilon_{\infty }+\frac{\varepsilon_{s}-\varepsilon_{\infty }}{1+i\omega \tau }=\varepsilon^{\prime }-i\varepsilon^{\prime \prime }$$

where $\varepsilon_{\infty }$ is the optical frequency dielectric constant; $\varepsilon_{s}$ is the relative dielectric constant in the static case; and *τ* is the relaxation time of the dielectric, determined by the structure of the dielectric and the ambient temperature, which determines the rate of increase of the dielectric polarization.

$\varepsilon^{\prime }$ and $\varepsilon^{\prime \prime }$ can be expressed as follows:

(4) $$\varepsilon^{\prime }=\varepsilon_{\infty }+\frac{\varepsilon_{s}-\varepsilon_{\infty }}{1+\omega^{2}\tau^{2}}$$

(5) $$\varepsilon^{\prime \prime }=\frac{{(\varepsilon }_{s}-\varepsilon_{\infty })\omega \tau }{1+\omega^{2}\tau^{2}}$$

The real part of the complex permittivity is the relative permittivity, and the imaginary part is the loss factor. $\varepsilon^{\prime }$ decreases with an increasing frequency. When the frequency is low, the value of $\varepsilon^{\prime }$ is equal to the static permittivity. When the frequency is large enough, the value of $\varepsilon^{\prime }$ is equal to the permittivity at infinity. When *ωτ* = 1, $\varepsilon^{\prime \prime }$ is maximum, and the frequency at this time is the critical frequency, and the dielectric exhibits a strong relaxation absorption of electromagnetic energy.

The relative permittivity of liquid water at room temperature in the terahertz band (0.1 THz–3 THz) is generally described by the double Debye model [53], i.e.,:

(6) $$\varepsilon_{w}=\varepsilon_{\infty }+\frac{∆\varepsilon_{1}}{1+i\omega \tau_{1}}+\frac{∆\varepsilon_{2}}{1+i\omega \tau_{2}}$$

where $\varepsilon_{w}$ is the relative permittivity of water; $\varepsilon_{\infty }$ is the DC relative permittivity; $∆\varepsilon_{1}$ is the constant of the slow relaxation process; $∆\varepsilon_{2}$ is the constant of the fast relaxation process; and $\tau_{1}$ and $\tau_{2}$ are the time constants of the slow and fast relaxation processes, respectively.

### 2.3. Effective Medium Theory Model

The effective medium theory (EMT) is mainly used to study the dielectric properties of composite media by calculating their relative dielectric constants and treating the whole composite media as one medium using the equivalent relative dielectric constants [52]. The EMT evaluates the effective dielectric function of the macroscopic uniform medium depending on the permittivity of the individual components and their respective volume fractions [54]. The effective medium theory is used to analyze the interaction of the terahertz electric field with the mixture sample, providing hypothetical expressions for calculating the macroscopic dielectric properties of multicomponent composite materials [55]. The commonly used effective dielectric theory models include the Maxwell Garnett (MG) model [56], the Bruggeman (BG) model [57], and the Landau–LifshitZ–Looyenga (LLL) model [58]. Among them, the BG model and the LLL model are widely used in the field of terahertz bio-detection [59].

The MG model is one of the earliest EMT models, which attempts to characterize the local electric field effect by studying the effective polarization rate of a doped complex with dielectric constant $\varepsilon_{p}$ in a vacuum environment. The equivalent dielectric constant $\varepsilon_{eff}$ for this nonuniform composite medium is seen as follows:

(7) $$\frac{\varepsilon_{eff}-1}{\varepsilon_{eff}+2}=f_{p}\frac{\varepsilon_{p}-1}{\varepsilon_{p}+2}$$

where $f_{p}$ is the percentage of volume occupied by the dopant.

If the main medium is not in a vacuum and is replaced by a medium with a dielectric constant $\varepsilon_{h}$, then the equivalent dielectric constant MG equation in a general sense is shown below:

(8) $$\frac{\varepsilon_{eff}-\varepsilon_{h}}{\varepsilon_{eff}+2\varepsilon_{h}}=f_{p}\frac{\varepsilon_{p}-\varepsilon_{h}}{\varepsilon_{p}+2\varepsilon_{h}}$$

The volume percentage of dopant $f_{p}$ is as follows:

(9) $$f_{p}=\frac{\left(\varepsilon_{eff}-\varepsilon_{h})(\varepsilon_{p}+2\varepsilon_{h}\right)}{\left(\varepsilon_{eff}+2\varepsilon_{h})(\varepsilon_{p}-\varepsilon_{h}\right)}$$

The BG model treats the substance as a discontinuous term consisting of a stack of two particles with dielectric constants $\varepsilon_{p}$ (dopant) and $\varepsilon_{h}$ (primary medium), avoiding the variations caused by high filling factors in the MG model. The BG model is widely used in highly doped mixtures, and the BG model equations are shown below:

(10) $$f_{p}\frac{\varepsilon_{eff}-\varepsilon_{p}}{\varepsilon_{eff}+2\varepsilon_{p}}+\left(1-f_{p}\right)\frac{\varepsilon_{eff}-\varepsilon_{h}}{\varepsilon_{eff}+2\varepsilon_{h}}=0$$

This gives the equivalent dielectric constant of the mixture:

(11) $$\varepsilon_{eff}=\frac{1}{4}(\beta +\sqrt{\beta^{2}+8\varepsilon_{h}\varepsilon_{p}})$$

where $\beta =\left(2-3f_{p}\right)\varepsilon_{h}+\left(3f_{p}-1\right)\varepsilon_{p}.$

The volume percentage of the dopants is as follows:

(12) $$f_{p}=1-\frac{\varepsilon_{p}-\varepsilon_{eff}}{\varepsilon_{p}-\varepsilon_{h}}{\left(\frac{\varepsilon_{h}}{\varepsilon_{eff}}\right)}^{\frac{1}{3}}$$

Unlike the MG model and BG model, the LLL model uses a different assumption with the advantage of not specifying the shape of the embedded particles and is theoretically applicable to mixtures of irregularly shaped particles. As with the BG model, the results are the same after exchanging the primary medium and dopant and can be generalized to an extended theoretical model for characterizing the dielectric properties of multicomponent mixtures. The LLL model equations are shown as follows:

(13) $$\sqrt[{3}]{\varepsilon_{eff}}=f_{p}\sqrt[{3}]{\varepsilon_{p}}+f_{p}\sqrt[{3}]{\varepsilon_{h}}$$

This leads to the equivalent dielectric constant of the mixture as a whole:

(14) $$\varepsilon_{eff}={\left(f_{p}\sqrt[{3}]{\varepsilon_{p}}+f_{p}\sqrt[{3}]{\varepsilon_{h}}\right)}^{3}$$

The volume percentage of the dopants is as follows:

(15) $$f_{p}=\frac{\sqrt[{3}]{\varepsilon_{eff}}-\sqrt[{3}]{\varepsilon_{h}}}{\sqrt[{3}]{\varepsilon_{p}}-\sqrt[{3}]{\varepsilon_{h}}}$$

While using the terahertz technique to detect samples with different water contents, the sample is considered a mixture of water and several other substances, and the effective dielectric properties of the sample are related to the dielectric properties and volume fraction of each component substance including water [60]. The equivalent dielectric properties of the sample can be calculated from the volume fraction of each component and the optical parameters, and similarly the volume fraction of each component can be calculated from the known equivalent dielectric constant as well as the dielectric constant of each component substance [61]. The results of using water as a primary medium and water as a dopant are different, and the results of the different models are assumed to be different, so it is important to choose the appropriate effective dielectric theory model.

### 2.4. Machine Learning Theory

Terahertz spectroscopy and imaging techniques are widely used in various fields for sample detection, and the acquired terahertz signals containing a large amount of sample information need to be analyzed in order to have a clearer understanding of the sample detection information. The combination of terahertz techniques with chemometric methods, machine learning, and search algorithms can effectively build analytical models of sample information [62]. In recent years, machine learning techniques have developed rapidly, and many new learning models and learning algorithms have emerged. Therefore, combination with state-of-the-art machine learning techniques enables more efficient data analysis for terahertz applications [63].

Machine learning is typically divided into three categories: supervised learning, unsupervised learning, and reinforcement learning. Supervised learning uses a training dataset consisting of many inputs and corresponding labels, with each label representing the target output for each input. Unsupervised learning uses an unlabeled training dataset, while reinforcement learning has no training dataset and uses a trial-and-error approach to learn the desired model. Several machine learning models that are commonly used for terahertz data analysis are described next [64].

Multiple linear regression refers to the analysis of the correlation between two or more independent variables and a dependent variable, and linear regression analysis means that there exists a linear relationship between the independent and dependent variables. MLR has the advantages of being simple and fast and having good interpretability, but it requires strict assumptions, a significant effect of the independent variable on the dependent variable, and a close linear relationship, as well as the presence of multicollinearity and other problems [65].

Principal component analysis is the most commonly used linear dimensionality reduction method for data, which maps high-dimensional data to low-dimensional space through some linear projection relationship, using fewer data dimensions to retain more original data characteristics. The PCA method, as an unsupervised learning method, is not limited by sample labels and can reduce the workload of indicator selection, but the interpreted meaning has some ambiguity and is less complete than the original data sample [66].

The partial least squares method, first proposed by S. Wold and C. Albano et al. in 1983, is a new multivariate statistical data analysis method, which can simultaneously implement MLR, PCA, and correlation analysis between two sets of variables. Although PCA solves the problem of the co-linearity of independent variables, it fails to fully consider the explanatory role of the principal elements of the independent variables on the variation of the dependent variable. The PLS method solves the problem of the co-linearity of independent variables by mapping the high-dimensional data space of the independent and dependent variables to the low-dimensional space and then establishing a univariate linear regression relationship between their eigenvectors, which not only solves the covariance problem but also removes the influence of useless noise on the regression and reduces the number of variables in the model [67].

Support vector machines (SVMs), initially proposed by Vapnik et al. in 1963, are a binary classification model for small and medium data samples and nonlinear and high-dimensional classification problems, and SVMs were considered one of the most successful and best-performing algorithms in machine learning in the last decade before the advent of deep learning [68].

## 3. Research Progress of Moisture Detection Based on THz Wave

### 3.1. Relationship between Water Molecular Network Dynamics and THz Spectra

Water is the dominant substance on the Earth’s surface and possesses an extremely complex network of hydrogen bonds in its molecules, with each water molecule forming an average of four hydrogen bonds in an almost tetrahedral configuration. The three-dimensional network of hydrogen-bonded water molecules contains complex intermolecular degrees of freedom with diverse dynamics, and the molecular dynamics (MD) associated with this network include restricted translational, rotational, and diffusive motions covering a very wide range of frequencies [69]. The complex network of hydrogen bonds in water molecules plays an important role in the thermodynamic properties of water; however, the rotation mode, vibration mode, and energy associated with hydrogen bonds of water molecules are in the terahertz band [70]. Most researchers have identified two relaxation modes of water: a slow relaxation in the GHz to 0.1 THz frequency range and a fast relaxation that occurs in the terahertz frequency range, on the sub-picosecond time scale [71]. In addition, water molecules, being polar molecules, have a strong resonant absorption of terahertz waves [72]. Therefore, it is necessary to study the relationship between the hydrogen bonding network dynamics of water and terahertz absorption spectra.

Elgabarty et al. [73] studied the dynamic energy flow in a liquid water hydrogen bonding network via pump–probe experiments, obtaining background-free bipolar signals of the trailing single exponential relaxation using an ultrathin sample cell window. Using complementary experiments, force field and molecular dynamics simulations, the relaxation is attributed to the translational motion of the molecule, revealing an initial coupling of the terahertz electric field to the rotational degrees of freedom of the molecule, whose energy is rapidly transferred to the restricted translational motion of the neighboring molecule within the excitation pulse duration. The collective rotational degrees of freedom of water are excited using a strong terahertz pulse resonance, and the optical anisotropy arising in the terahertz Kerr effect (TKE) is explored. The temperature-dependent TKE of liquid water, the TKE of water vapor, and the optical Kerr effect (OKE) of liquid water are experimentally probed, and the AIMD and force-field-based FFMD simulations are compared under the same terahertz field to gain insight into the intermolecular energy transfer process in water.

Zhao et al. [74] used a strong and ultrabroadband THz pulse resonance to excite the intermolecular modes of liquid water and observed transient birefringence signals induced by bipolar THz fields in a free-flowing water film. The decomposition of the bipolar signal into a positive signal induced by hydrogen bond stretching vibrations and a negative signal induced by hydrogen bond bending vibrations indicates that the polarizability perturbation of water exhibits competing states under stretching and bending conditions. A resonant oscillator model of the hydrogen bond network related to the dielectric sensitivity is proposed and combined with the Lorentzian kinetic equations to study the dynamics and intermolecular structure of liquid water. The authors suggest that the transient rotation of molecules generates an induced dipole moment that transfers the THz-field-driven momentum to the confined translational motion of neighboring water molecules, which leads to components of polarizability anisotropy perpendicular and parallel to the hydrogen bonds separately, resulting in bidirectional properties. The Kerr coefficient equation associated with the intermolecular mode of water was established, and the measured TKE response showed a significantly decaying oscillatory process with a double-peaked time interval of 170 fs, indicating that this phenomenon cannot be explained by a fast relaxation process. The sub-picosecond time scale of 170 fs is consistent with the characteristic frequency of the hydrogen bond stretching vibration, indicating that the positive response of the Kerr effect originates mainly from the stretching vibration rather than the fast relaxation process. The negative response exhibits a sub-picosecond scale, and theoretical simulations show that this feature mainly originates from the damped oscillation process of the bending mode rather than the molecular orientation process.

**Figure 2:**
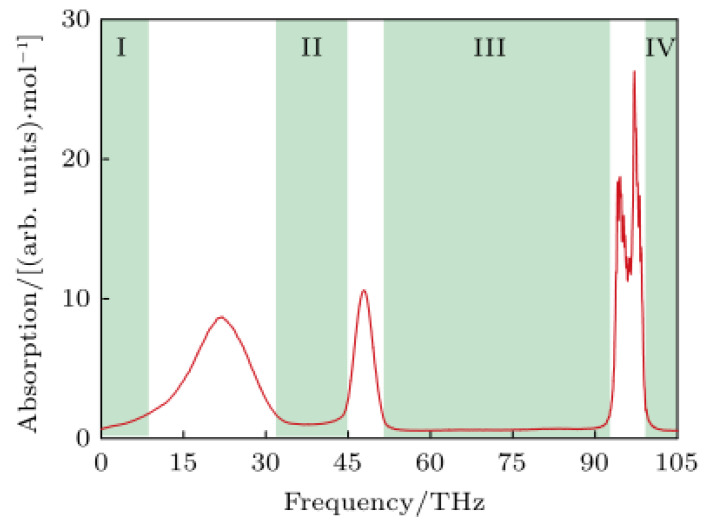
Terahertz absorption spectrum of water [76].

Cai et al. [75] used a terahertz method combined with microfluidics to study deionized water treated with an electric field and showed that the intensity of the absorption of terahertz waves by deionized water varied with the time of the electric field treatment. It is inferred that the application of an applied electric field to water molecules causes a change in the dipole moment of water molecules, which has an impact on the rotation and vibration of the overall water molecules, changing the network structure of hydrogen bonds and leading to an enhancement of the spectral intensity. Duan et al. [76] indicated the existence of three main peaks in the terahertz band for water, as shown in Figure 2. The first peak corresponds to the vibration mode of the hydrogen bond network in water and is located at 5 THz–30 THz; the second peak corresponds to the bending vibration of the bond angle inside the water molecule and is located at 45 THz–50 THz; the third peak corresponds to the stretching vibration of the bond length inside the water molecule and is located at 90 THz–105 THz. The terahertz absorption intensities of different water models (SPC, SPC/E, TIP3P, TIP4P, and TIP4P-Ew) at room temperature and pressure were investigated using molecular dynamics simulations, and it was found that all the different water models qualitatively described the various vibrational modes of bulk-phase water. The relationship between the THz absorption spectra and the hydrogen bonding network of water molecules under the influence of temperature was further investigated, and it was found that the THz spectra of the hydrogen bonding network would be red-shifted when the temperature increased, indicating a strong correlation between its center frequency and the strength of the network.

Rasekh et al. [77] used terahertz techniques to study the nonlinear absorption spectra of water vapor and attributed this nonlinear response to step multiphoton jumps in water molecules. Wang et al. [78] characterized the water-involved hydrogen bonding in oil-paper insulating materials via terahertz dielectric spectroscopy. A relaxation resonance integral polarization model of the water-involved hydrogen bonding effect was developed to describe the dielectric constant of oil-paper insulating materials under the action of water. Zhang et al. [79] used two-dimensional terahertz rotational spectroscopy to observe the correlation between higher-order rotational coherence and rotational leap, and the results demonstrated the sensitivity of rotational correlations measured in two-dimensional terahertz spectroscopy to water vapor molecular interactions and complexation. Shiraga et al. [80] used broadband terahertz and low-frequency Raman spectroscopy to detect intermolecular stretching patterns in liquid water, respectively, and the terahertz spectrum was able to detect significant redshifts and broadening compared to the Raman spectrum. Yada et al. [81] used a terahertz time-domain decay total reflection system to determine the dielectric constants of water and heavy water, which were decomposed into four components: slow relaxation, fast relaxation, intermolecular stretching vibrations, and intermolecular calibration. The temperature dependence and isotopic shifts of the fast relaxation are consistent with the single-relaxation model. In the dynamic hydrogen bonding network, the short-lived structures on sub-picosecond time scales indicate the heterogeneity of the water structure. Penkov et al. [82] used terahertz to study the hydrated shell layer of adenosine-5’-triphosphate (ATP) in water and MgCl2 solutions using the terahertz time-domain spectroscopy technique and dynamic light scattering. The distorted water structure in aqueous ATP solutions was revealed by THz spectroscopy, showing tightly bound water molecules. When ATP is bound to Mg ions, an aligned structure with an increased number of hydrogen bonds is observed. The increase in the shell size is explained by the scattering properties by the formation of a layer with a refractive coefficient similar to that of water. Bozorova et al. [83] presented molecular dynamics simulations of THz spectra of aqueous acetic acid solutions, where the absorption spectra of aqueous acetic acid solutions were calculated in the THz range, showing that the collective dynamics of water molecules depended on the acetic acid concentration.

As a polar molecule, water has a complex hydrogen bonding network, and the time required for its disruption and rearrangement operations during molecular thermal motion are in the picosecond and sub-picosecond orders, and the corresponding electromagnetic wave frequencies are located in the terahertz band, so water molecules are extremely sensitive to terahertz waves and have an extremely strong absorption effect. For that reason, terahertz are extremely important for studying the dynamic processes of water networks.

### 3.2. Terahertz Detection of Moisture Content

#### 3.2.1. Terahertz Technique to Detect Free Water

Water content is one of the important indicators of crop quality, and too much or too little moisture can affect the quality of crops [84]. The water content of plants varies greatly during different growth cycles, and the nondestructive detection of their water content using terahertz technology allows for an understanding of the growth dynamics of plants by monitoring the water content in their leaves and also by studying the effect of other factors on the variation in the water content in plants.

Chua et al. [85] 2005 first used the THz-TDS transmissive mode to measure the absorption spectra of wheat particles in the frequency range of 0.1 THz–2.0 THz, subtracting the spectra of wet wheat samples from those of dried wheat samples to investigate the relationship between absorption and moisture content. Ma et al. [86] used the transmissive mode of the terahertz time-domain spectroscopy system to detect gastrodia elata samples with different moisture contents and calculate their absorption coefficients to obtain the relationship between absorption coefficients and moisture content, thus enabling the detection of pharmaceuticals with different moisture contents. Gente et al. [87] 2015 discussed the possibility of terahertz time-domain spectroscopy systems for the detection of moisture content and in 2018 [88] proposed a portable compact system based on THz-QTDS using a multimode laser diode to generate a light beat signal of light mixing to produce THz radiation, which was used to monitor the moisture status of corn plants in outdoor fields. Horita et al. [89] developed a novel nondestructive detection method based on a THz-ATR system for measuring the optical constants of liquids circulating on the surface of an ATR prism. It was shown experimentally that the moisture content of ethanol could be determined by monitoring the refractive index of the liquid in the THz range, and the proposed system was able to successfully assess ethanol concentrations and moisture contents.

Zahid et al. [90] et al. detected the water content and characteristics in plant leaves by absorption spectra of water molecules at THz frequencies for four consecutive days, and based on the calculated dielectric constants, they inferred that leaf samples became increasingly transmissive to THz waves over time, while a loss of leaf weight and thickness was observed, and morphological changes occurred in fresh and water-stressed leaves. Bin Li et al. [91] used the scattering effect at terahertz frequencies to monitor the water status of leaves, using reflection and transmission modes for plant leaves with different water contents, respectively, and proposed to combine integral equation (IEM) and radiative transfer theory (RTE) models to study the interaction between THz and plant leaves. Then, they investigated the reflection and transmission geometries of THz at different temperatures, salinities, and polarization conditions. Ran Li et al. [92] used tunable THz radiation to measure leaf water contents, using the differential absorption characteristics of THz radiation at multiple frequencies within plant leaves to determine the absolute water content in real time, and combined the THz system with a wet and dry meter to generate a pressure-volume (PV) curve for determining leaf tissue water relationship parameters, which provided repeatable, nondestructive measurements of leaf water content. The efficiency of leaf PV curve generation was improved by providing repeatable, nondestructive measurements of leaf water content and reducing user processing time. Kumar et al. [93] used broadband THz-TDS to sense diatoms by detecting the intensity of water absorption in the THz range caused by the interaction between water and diatoms. Santesteban et al. [94] used the THz-TDS reflection mode to detect the water content of grapevine trunks and calculated the reflection coefficients to assess the applicability of THz reflection properties in plant water status estimation through three experiments: watering cycles, the coupling of THz signals with light and dark changes, and the coupling of THz signals with xylem and bast activity. Hernandez et al. [95] used THz-TDS combined with the effective medium theory to simultaneously quantify the water content and thickness of leather. Dong et al. [96] accurately represented the trace moisture in epoxy resin via the established terahertz time-domain spectroscopy system, which effectively shortened the detection time of the epoxy resin water content.

#### 3.2.2. Terahertz Technology to Detect Combined Water

Water in nature generally exists in two forms: free water, which is a freely moving water molecule that can be used as a good solvent and as a medium for biochemical reactions, and bound water, which exists as OH^−^, H^+^, and H_3_O^+^ and is a hydrated water molecule that is adsorbed by colloidal particles and macromolecules in cells or exists in the space of macromolecular structures and neither dissolves other substances nor participates in metabolism [97]. Different states of water respond to THz with different values and it is necessary to use terahertz techniques for the detection of bound water in biological objects.

**Figure 3:**
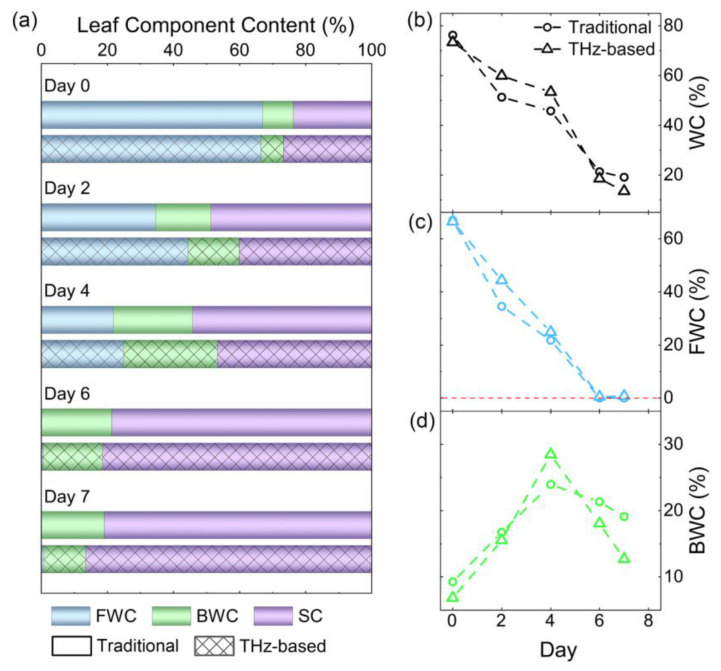
The results of free and bound water detection in leaves with different air-drying times via conventional method and THz spectroscopy. (**a**) is the percentage of free water content, bound water content, and solid matter content. (**b**–**d**) are the results of the variation in moisture content, free water content, and bound water content with drying time [103].

Cherkasova et al. [98] used THz spectroscopy to characterize the transformation of water in glucose from free water to bound water by measuring the THz spectra of samples in three states: dry glucose, wet glucose (suspension), and an aqueous glucose solution, and extracting the absorption and refraction spectra of bound water in the range of 0.07 THz–2.6 THz in the wet glucose powder (no free water contribution) state. The bound water was found to be one order of magnitude weaker than the free water. A two-component relaxation model was used for free water and bound water to describe the dielectric functions of low concentration glucose aqueous solutions in the frequency range of 0.1 THz–5 THz, and it was found that the relaxation time of bound water was 30 times higher than that of free water. The LLL model from the effective medium theory was used to describe the dielectric constants of the total composite medium with four components (free water, bound water, dissolved crystals, and dry crystals of glucose), each with a specific spectral response. Hoshina et al. [99] studied the bound water information of polyethylene–vinyl alcohol copolymer (EVOH) films with different humidities via THz absorption spectroscopy and observed the intermolecular stretching pattern of bound water at about 6 THz. The bound water was classified into three types by generalized two-dimensional correlation spectroscopy (2DCOS) and perturbation-related two-dimensional moving window (PCMW2D) correlation spectroscopy: frozen water with an amorphous structure, liquid water with translational motion, and nearest-neighbor water with a weaker hydrogen bonding network. Devi et al. [100] measured two types of proton exchange membranes (PEMs), hydrated Nafion and sulphonated Polyether Ether Ketone (sPEEK) membranes, using THz spectroscopy, and found that sPEEK has a higher water absorption capacity than Nafion. The proportions of volumetric water, bound water, and free water with time were estimated by fitting the complex dielectric constant using a double Debye model, and sPEEK was found to have a higher proportion of bound water than Nafion. Penkova et al. [101] studied DNA hydration shells in solutions via terahertz time-domain spectroscopy, describing the dielectric constants of three DNA solutions using an effective medium model and calculating even relaxation parameters related to bound and free water and intermolecular oscillations. Charkhesht et al. [102] used nodal spectra in the frequency range of 50 MHz–0.5 THz to describe the relaxation kinetics of glycerol and used the Debye relaxation model to determine the structure of the hydration shell around the glycerol molecule and the kinetics of bound water. The experimental results showed that the dielectric response of the mixture was controlled by free water, bound water, and glycerol when the glycerol concentration was below 7.5 mol%. At higher glycerol concentrations, the hydration shell layers start to merge and overlap, and the dielectric response from the bound water shows saturation behavior, where the dielectric response increases with an increasing glycerol concentration. Zang et al. [103] used terahertz spectroscopy combined with the effective medium theoretical model for the quantitative detection of free and bound water in plant leaves, using leaf samples of four different species and the particle swarm optimization algorithm for spectral analysis at 0.7 THz and 0.9 THz, and the results showed that the quantitative detection efficiency could be significantly improved. The results of free and bound water detection in different air-dried leaves using the conventional and THz methods, respectively, are shown in Figure 3, with good agreement between the two methods.

#### 3.2.3. Terahertz Technology Combined with Machine Learning Method to Detect Moisture Content

With the rapid development of terahertz technology, the means of moisture content detection have been supplemented and the prediction and quantitative analysis of moisture content has been further developed. The detection of sample moisture content using terahertz spectroscopy, combined with machine learning methods MLR [65], PCA [66], PLS [67], and SVM [68] to establish a prediction model between terahertz optical parameters of samples and sample moisture content, can provide rapid prediction of moisture content [104,105].

**Figure 4:**
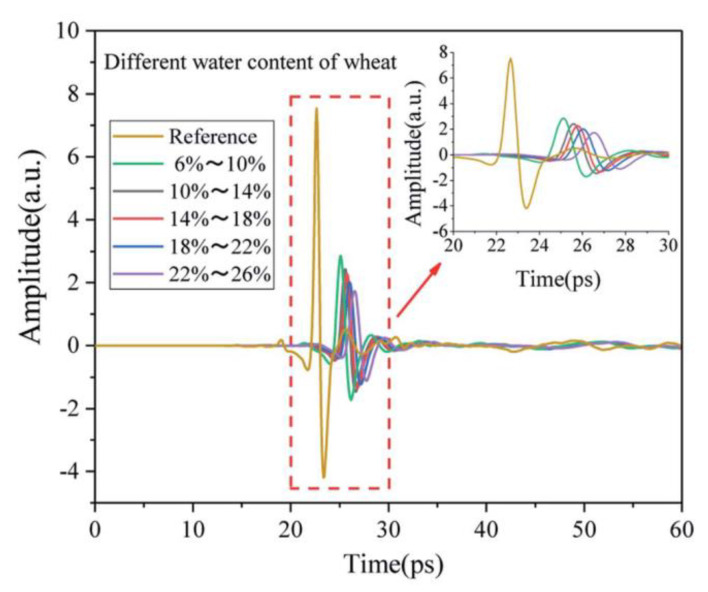
THz time-domain spectra of wheat with different moisture levels [107].

Ren et al. [106] used THz waves and the Swissto12 Material Characterization Kit (MCK) to monitor changes in moisture content in fruits in the frequency range of 0.75 THz–1.1 THz. Features were extracted from the temporal, frequency, and time-frequency domains using three machine learning methods (SVM, KNN, and D-Tree) to accurately evaluate the moisture content in apple and mango slices. The results showed that SVMs outperformed the results of other classifiers using 10-fold validation and cross-validation techniques that retained one observation, with 100% accuracy for all three classifiers on days 1 and 4 for 80% and 2% moisture contents in the two fruit slices, respectively, and 95% accuracy for the intermediate moisture content in the two fruit slices for days 2 and 3. Shen et al. [107] developed a prediction model based on the THz spectrum and absorption coefficient spectrum of wheat with different moisture levels. The THz time-domain spectra of wheat samples with different moisture contents are shown in Figure 4. Compared with the reference signal, wheat samples with different moisture contents showed regular time delays, and different moisture contents affected the propagation speed of the terahertz waves in the samples. As the moisture content increases, the absorption of THz waves in wheat samples gradually increases, resulting in a gradient decrease in the peak signal and a gradient decrease in the spectrum. The collected THz spectra were pre-processed via SG, MSC, and SNV, feature extraction was performed using the TS forbidden search algorithm, and finally a quantitative prediction model of wheat moisture was established using PLS. The results showed that SG + MSC + TS + PLS modeled the absorption coefficient spectra best, with Rc and RMSEC reaching 0.9522 and 0.4730 for the calibration set and Rp and RMSEP reaching 0.9531 and 0.5396 for the prediction set.

Cao et al. [108] used THz-ATR to test five water samples, such as pure water, tap water, and river water, and used PCA to downscale and extract features for their refractive indices, input the processed data into a SVM to construct a classification model for water samples, and use three algorithms, GridSearch, particle swarm (PSO), and the genetic algorithm (GA), to optimize the SVM parameters. The results show that the accuracy of the SVM classification models constructed by different optimization algorithms is above 99.0%, and the PCA-SVM classification model constructed based on a refractive index using the particle swarm optimization algorithm has the best result. Chen et al. [109] used terahertz spectroscopy combined with a characteristic spectral region screening algorithm to quantify the water content of engine lubricating oil by obtaining absorption coefficient spectra in the transmission mode, and the pre-processed spectra were screened for characteristic spectral regions using three PLS methods: regular interval partial least squares (iPLS), backward interval partial least squares (BiPLS), and joint interval partial least squares (SiPLS) for THz spectra, respectively. The results show that the best modeling results are achieved when using the BiPLS model applied to the quantitative analysis of lubricant moisture content. Wu et al. [110] used the same feature spectral region screening methods (mwPLS, iPLS, BiPLS, and SiPLS) for the feature screening of the moisture absorption coefficient spectra of maize seeds measured using THz-ATR and finally used RBF-based support vector machine SVR and grid search methods to construct a moisture content prediction model. Li et al. [111] established PLS and MLR prediction models based on THz time-domain spectral maxima, minima, and full spectra, as well as absorption coefficient spectra and refractive index, respectively, for the prediction and analysis of soybean leaf water contents, in which the PLS model based on THz time-domain spectral extremes predicted the best results. Zhang et al. [112] used partial least squares regression (PLSR) to construct a model for quantitative analysis and the detection of the moisture content of lettuce based on THz spectra. Huang et al. [113], who detected the moisture content in cattle feed based on THz and near-infrared (NIR) spectra, constructed prediction models based on head-to-tail spliced fused spectral data and spectral feature variables fused in the feature layer combined with PLSR, respectively, where the Rp, RMSEP, and RPD of the fused spectral data in the feature layer reached 0.9933, 0.0069, and 8.7386, with better results. The results indicated that the fusion of THz and NIR spectral data could predict the feed moisture content more accurately than the prediction models built by single THz and NIR.

### 3.3. Moisture Distribution Visualization Study

The spatial distribution of water in a substance, to some extent, can reflect the local lesion, damage, and health status of the tested substance, and the visualization of the spatial state of water in substances in different environments can enable the study of water transport mechanisms and physiological activities [114]. The visualization of water distribution using terahertz spectral imaging is mainly applied to the detection of the water distribution in plant leaves as a way to characterize the physiological state of plants.

**Figure 5:**
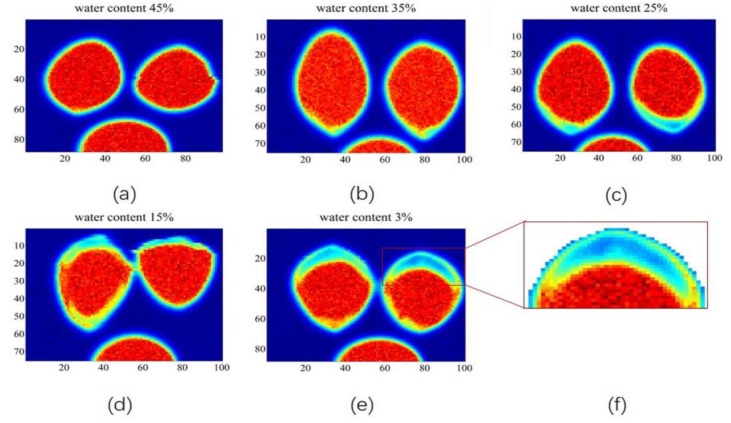
THz images of intact seeds of Ginkgo biloba fruits with different water contents. (**a**) water content 45%, (**b**) water content 35%, (**c**) water content 25%, (**d**) water content 15%, (**e**) water content 3% and (**f**) a enlarged region of interest [116].

**Figure 6:**
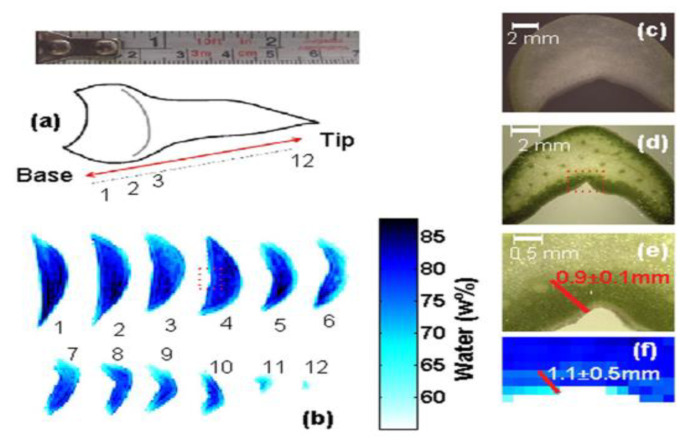
Water distribution at different locations of cross-sections of agave leaves. (**a**) Approximate positions of sections 1–12 over the length of an agave leaf. (**b**) Tomographic sections showing the water content distribution of an agave leaf. (**c**) Optical microscopy images of an agave basal (white) leaf section at 8×. (**d**,**e**) Bright light microscopy images of an agave middle (green) leaf section at 8× and 35× magnification respectively. The red box in (**d**), indicates the enlarged region shown in (**e**). (**f**) Enlarged terahertz image corresponding the box area of section 4 in (**b**) [120].

Lei et al. [115] used a terahertz time-domain imaging system combined with a deep learning approach (AE-GAN) to visualize the moisture distribution of sunflower seeds and automatically learned kernel information from the spectral potential representation of intact seeds through adversarial learning to achieve feature separation and successfully obtained high-quality chemical distribution maps of the energy and moisture content within the sunflower seed shells. Gong et al. [116] used terahertz spectroscopy and imaging techniques to study the variation in the moisture content of ginkgo nuts, characterizing the effect of moisture on THz using the time-domain spectrum and frequency signal of the absorption coefficient, and the variation in ginkgo nuts at different moisture contents (3%, 15%, 25%, 35%, and 45%) using terahertz imaging techniques. The imaging results are shown in Figure 5. When the seed moisture content is 45%, there is almost no gap between the seed and the shell, and as the seed gradually dehydrates, the seed kernel starts to shrink and a gap appears between itself and the seed shell. Shchepetilnikov et al. [117] used a terahertz imaging system to quantify the temporal evolution and spatial distribution of the water content in clover leaves, and the experimental results showed that the TeraSense imager can be effectively used to monitor the hydration status of plants in their natural environment. Bensalem et al. [118] demonstrated the possibility of using THz imaging techniques to measure the moisture distribution of wood samples during the drying process. Bu et al. [119] extracted terahertz images of soybean leaves in three feature bands of 0.577, 1.098, and 1.163 THz, dividing them into leaf vein images and leaf flesh images using adaptive threshold segmentation, and used the image gray-scale mean values of the three types of images including leaf images as feature parameters to build soybean leaf moisture prediction models based on PLSR, MLR, and other methods, respectively. The results showed that LS-SVM prediction based on the gray-scale features of leaf flesh images was the best. Singh et al. [120] initially used THz tomography to determine the water content of agave leaf cross-sections. Considering the agave leaf as a mixture of water, dry matter, and air, the effective medium theoretical model was used to fit the transmission spectrum of each pixel of the terahertz image according to the LLL model, and then the water content was estimated pixel by pixel from the terahertz image. As shown in Figure 6, the images were able to clearly show the different water contents in the exterior and interior of the leaf, and it was possible to observe the low water content layer in the exterior of the leaf and the more watery portion in the interior, with the water content showing an additional degree of variation in the leaf cross-section and gradually decreasing along the leaf axis.

## 4. Challenges and Outlook

### 4.1. Challenges

#### 4.1.1. Strong Absorption of Terahertz Waves by Water

Water is extremely sensitive to terahertz waves and has a strong absorption effect, but the reasons for this have not been fully explained. The response characteristics of water to terahertz waves that can be detected at present are still in the weak absorption window and cannot be fully explained for their mechanism of action. Water also has a strong absorption window in the terahertz band, and some spectral features in this window can complement the interaction of water with terahertz. For samples with high water contents, the direct observation of the response characteristics of other substances in the sample to terahertz waves can be affected when using terahertz techniques for their detection without drying. In addition, different states of water have different mechanisms of action on terahertz waves and different spectral characteristics, such as free and bound water, water in normal and cancerous tissues, etc.

#### 4.1.2. Water Migration Process

Water exists in all living organisms, and changes in its content as well as the water distribution in living organisms reflects changes in the characteristics of the organisms themselves to some extent. The water status in the organism changes with the time and environment, such as water migration in plants during the process of water intake and transport from roots to leaves and other parts; water migration in wheat seeds during the process of water intake and penetration from epidermis and embryo to endosperm; water migration in the process of developing normal tissues into cancerous tissues; and the process of free water to bound water when the environment changes. The mechanism principle of the water migration process is unclear, and most of the current applications of water content detection using terahertz technology are for a specific water content at a specific time, while the dynamic migration process of water content changes in organisms using terahertz technology is not sufficiently explained.

#### 4.1.3. Prediction of Moisture Content and Moisture Distribution

The accurate prediction of moisture conditions is beneficial to accurately grasp the growth condition of organisms and the operation of tissues and organs and to accurately control the quality of food grains. With the development of artificial intelligence, traditional machine learning methods have been used for moisture detection via terahertz technology, and terahertz technology combined with machine learning methods can achieve moisture content prediction, but its prediction accuracy still needs to be improved. In addition, the accurate control of biological moisture distribution is beneficial to the study of various mechanistic characteristics of organisms. At present, there are few studies on the prediction of moisture distribution via terahertz imaging technology combined with artificial intelligence methods.

### 4.2. Outlook

#### 4.2.1. THz Combined with Dielectric Mixture Theory to Improve the Detection of Water Molecules

The strong absorption of terahertz waves by polar molecules, such as water, has led to the detection of these substances via terahertz techniques, but this has been a challenge. The dielectric mixture theory provides a physics-based approach to explain most of the factors affecting the measurement of the dielectric constant of water molecules, including the water state, grain density, temperature, etc. The THz technique combined with the dielectric mixture theory can estimate the dielectric constant of water molecules more accurately, thus improving the detection capability of THz for water molecules. In future, it is of necessity to combine the new generation of theoretical models, such as dielectric mixture theory, to explain the strong absorption of terahertz waves by water in a clearer and more understandable way through simulations and so on. The detection capability of terahertz needs to be improved to achieve a strong absorption window detection of water in the terahertz band, so that the interaction between water and terahertz waves can be more fully explained. Further research is needed to study the interaction law between water and terahertz waves in different states and environments, so that the rapid response of water in the terahertz band can be more fully described.

#### 4.2.2. Combining AI to Improve the Real-Time Monitoring of the Water Migration Process via THz

Many researchers have already shown the water distribution of plant leaves using imaging. In future, more research is needed to study the water migration process of biological tissues such as plant leaves to describe the changes of water distribution more clearly, so as to better explain the causes of changes in biological mechanisms. The corresponding model algorithm needs to be soundly improved and combined with AI and other technologies to make the terahertz technology provide a reasonable explanation of the mechanism principle of water migration. By reflecting the migration process of moisture content through the real-time changes in terahertz spectra, the factors affecting the changes in the moisture content in organisms and the interactions of substances in organisms can be explained more reasonably.

#### 4.2.3. Combining THz + Deep Learning

The prediction accuracy of moisture content still needs to be improved, and the prediction of moisture distribution also needs to be studied in depth. With the rapid development of artificial intelligence, the development of deep learning is becoming more and more mature, and the rapidly developing deep learning methods have a stronger expression ability compared with the traditional machine learning methods. At present, most of the methods used for moisture prediction are still traditional machine learning methods. Thus, more research is needed on the prediction of moisture content by combining terahertz technology with deep learning methods to improve the accuracy of moisture content prediction in the near future. Since deep learning has a strong two-dimensional image processing capability, we need to study the prediction of moisture distribution by combining terahertz imaging technology with deep learning methods in depth.

## 5. Conclusions

The strong absorption of terahertz radiation by water as a polar molecule makes the terahertz technique a powerful tool for water detection in samples. The use of terahertz spectroscopy and imaging techniques to measure and characterize the water content and water distribution, respectively, allows for more in-depth studies due to a greater understanding of the water state in samples. However, there are still some problems, such as the low resolution of water detection by terahertz and incomplete explanation of water the migration process, which need to be solved by more effective methods. Research progress shows that terahertz technology has great potential for water detection, and the related theoretical methods can be applied to other fields. The rapid development of terahertz technology proves its potential advantages in moisture detection applications and provides novel technological solutions with promising applications in fields such as agriculture and biochemistry.

This paper reviews the current research development of moisture detection using terahertz technology. Firstly, this paper introduces the theory of terahertz technology for moisture detection, outlining the relaxation model, effective medium theory, and other theoretical methods and models related to moisture detection and compares the relationship between the water molecular network and terahertz spectrum to explain water’s strong absorption of terahertz. Secondly, we summarize the research progress of the application of terahertz technology for moisture content and moisture distribution detection, respectively. Finally, the challenges and outlook of the application related to the terahertz wave detection of water are discussed to provide innovative value for the development of terahertz technology in the field of moisture detection.

## Figures and Tables

**Table 1:** Comparison of some terahertz imaging techniques.

Target	Passive Imaging	Active Radar Imaging	Time-Domain Spectral Imaging	Near-Field Super-Resolution Imaging
Cost	low	high	high	high
Signal-to-noise ratio	low	high	high	medium
Radiation source	no	yes	yes	yes
Resolution ratio	lower	higher	high	very high
Real-time imaging	easier to achieve	difficult to achieve	difficult to achieve	difficult to achieve
3D imaging	no	yes	yes	yes
Imaging algorithm	relatively simple	relatively complex	relatively simple	relatively simple
Disturbed by the environment	easily	not easily	easily	not easily
Technology maturity	high	medium	high	low

**Table 2:** Mainstream terahertz systems for moisture detection.

System Name	Abbreviation	Characteristic
Terahertz time-domain spectroscopy [38,39]	THz-TDS	High signal-to-noise ratio, good stability, wide bandwidth, and high sensitivity
Continuous-wave terahertz spectroscopy [40,41,42]	CW-THz	Low cost, single frequency, and suitable for spectral single frequency point acquisition
THz quasi time-domain spectroscopy [43,44]	THz-QTDS	Compact and low cost but limited signal bandwidth
Terahertz Attenuated Total Reflection [45,46,47,48]	THz-ATR	Suitable for samples with a high water content and strong absorption of terahertz waves

## Data Availability

All data generated or analyzed during this study are included in the manuscript.
